# Spatial and temporal proteome dynamics of glioma cells during oncolytic adenovirus Delta-24-RGD infection

**DOI:** 10.18632/oncotarget.25774

**Published:** 2018-07-24

**Authors:** Andrea González-Morales, Aintzane Zabaleta, Elizabeth Guruceaga, Marta M. Alonso, Marc García-Moure, Joaquín Fernández-Irigoyen, Enrique Santamaría

**Affiliations:** ^1^ Clinical Neuroproteomics Group, Navarrabiomed, Complejo Hospitalario de Navarra (CHN), Universidad Pública de Navarra (UPNA), Irunlarrea, Pamplona, Spain; ^2^ IDISNA, Navarra Institute for Health Research, Pamplona, Spain; ^3^ Proteored-ISCIII, Proteomics Unit, Navarrabiomed, Complejo Hospitalario de Navarra (CHN), Universidad Pública de Navarra (UPNA), Irunlarrea, Pamplona, Spain; ^4^ Oncohematology Area, University Hospital of Navarra, Center for Applied Medical Research, CIBERONC, Pamplona, Spain; ^5^ Bioinformatics Unit, Center for Applied Medical Research, University of Navarra, Pamplona, Spain; ^6^ Program in Solid Tumors and Biomarkers, Foundation for the Applied Medical Research, Pamplona, Spain; ^7^ Department of Pediatrics, University Hospital of Navarra, Pamplona, Spain

**Keywords:** adenovirus, Delta-24RGD infection, proteomics, glioma

## Abstract

Glioblastoma multiforme (GBM) is the most common and aggressive type of malignant glioma. Oncolytic adenoviruses are being modified to exploit the aberrant expression of proteins in tumor cells to increase the antiglioma efficacy. E1A mutant adenovirus Delta-24-RGD (DNX-2401) has shown a favorable toxicity profile and remarkable efficacy in a first-in-human phase I clinical trial. However, the comprehensive modulation of glioma metabolism in response to Delta-24-RGD infection is poorly understood. Integrating mass spectrometry based-quantitative proteomics, physical and functional interaction data, and biochemical approaches, we conducted a cell-wide study of cytosolic, nuclear, and secreted glioma proteomes throughout the early time course of Delta-24-RGD infection. In addition to the severe proteostasis impairment detected during the first hours post-infection (hpi), Delta-24-RGD induces a transient inhibition of signal transducer and activator of transcription 3 (STAT3), and transcription factor AP-1 (c-JUN) between 3 and 10hpi, increasing the nuclear factor kappa B (NF-κB) activity at 6hpi. Furthermore, Delta-24-RGD specifically modulates the activation dynamics of protein kinase C (PKC), extracellular signal–regulated kinase 1/2 (ERK1/2), and p38 mitogen-activated protein kinase (p38 MAPK) pathways early in infection. At extracellular level, Delta-24-RGD triggers a time –dependent dynamic production of multitasking cytokines, and chemotactic factors, suggesting potential pleiotropic effects on the immune system reactivation. Taken together, these data help us to understand the mechanisms used by Delta-24-RGD to exploit glioma proteome organization. Further mining of this proteomic resource may enable design and engineering complementary adenoviral based-vectors to increase the specificity and potency against glioma.

## INTRODUCTION

Glioblastoma multiforme (GBM) is the most common and aggressive type of malignant glioma, characterized by infiltrative growth that cause progressive neurologic dysfunction [1]. The standard treatment confers the patients a median overall survival time of 15 months [2], due to tumor cells that survive initial chemo- and radiotherapy causing tumor regrowth/recurrence. Thus, it is critical that the development of new therapies or the improvement of actual drugs have a positive impact on the course of this aggressive disease.

A great effort is devoted to understanding the biology of glioma cells to develop treatment strategies against their molecular defects [3]. One of these approaches is oncolytic virotherapy, which uses replication-competent viruses to destroy cancer cells [4, 5]. Oncolytic adenoviruses are being modified to exploit the aberrant expression of proteins in tumor cells to enhance tumor tropism and glioma-selective replication [6]. In particular, two stable genetic changes in the adenovirus genome were engineered, originating the E1A mutant adenovirus Delta-24-RGD, that replicates selectively in retinoblastoma (Rb) pathway deficient cells and infects tumor cells efficiently [7–9]. In general, results from pre-clinical and clinical studies have indicated that the adenovirus Delta-24-RGD is particularly attractive for malignant gliomas [9–13]. In addition, the combination of Delta-24-RGD with chemotherapy produced synergistic anti-glioma effects [14–16]. Interestingly, a recent clinical trial of Delta-24-RGD in patients with glioblastoma demonstrated favorable toxicity profile and remarkable clinical efficacy [17, 18]. So far, other trials for Delta-24-RGD (DNX-2401) in combination with IFN-gamma, temozolomide or anti-PD1 antibody are currently active (Clinical-Trials.gov identifiers NCT02197169, and NCT02798406).

It is well known that for an efficient cell lysis and adenoviral spread, Delta-24-RGD induces massive autophagy [19–22], a late response specifically regulated, in part, by the C-Jun N terminal kinases [23]. Understanding the cellular mechanisms that orchestrate the glioma cell response to oncolytic Delta-24-RGD virus will aid to develop novel vectors with enhanced capability to release viral progeny and, as a result, to elicit a more potent oncolytic effect. One of the potential avenues to increasing Delta-24-RGD potency that remains understudied is the monitoring of cellular intermediates underlying the glioma cell response prior to the activation of the autophagic process. In order to characterize the missing links in the biochemical understanding of the signaling pathways impaired in glioma cells during early phases of Delta-24-RGD infection, we have used a discovery platform combining a subcellular mass spectrometry based-quantitative proteomics approach, physical and functional interaction data, and biochemical approaches. The integration of these data will allow us to uncover means by which the molecular pathways are chronologically regulated during Delta-24-RGD infection at early time points.

## RESULTS

### Characterization of the early proteostasis impairment induced by Delta-24-RGD infection in glioma cells

**Figure 1:**
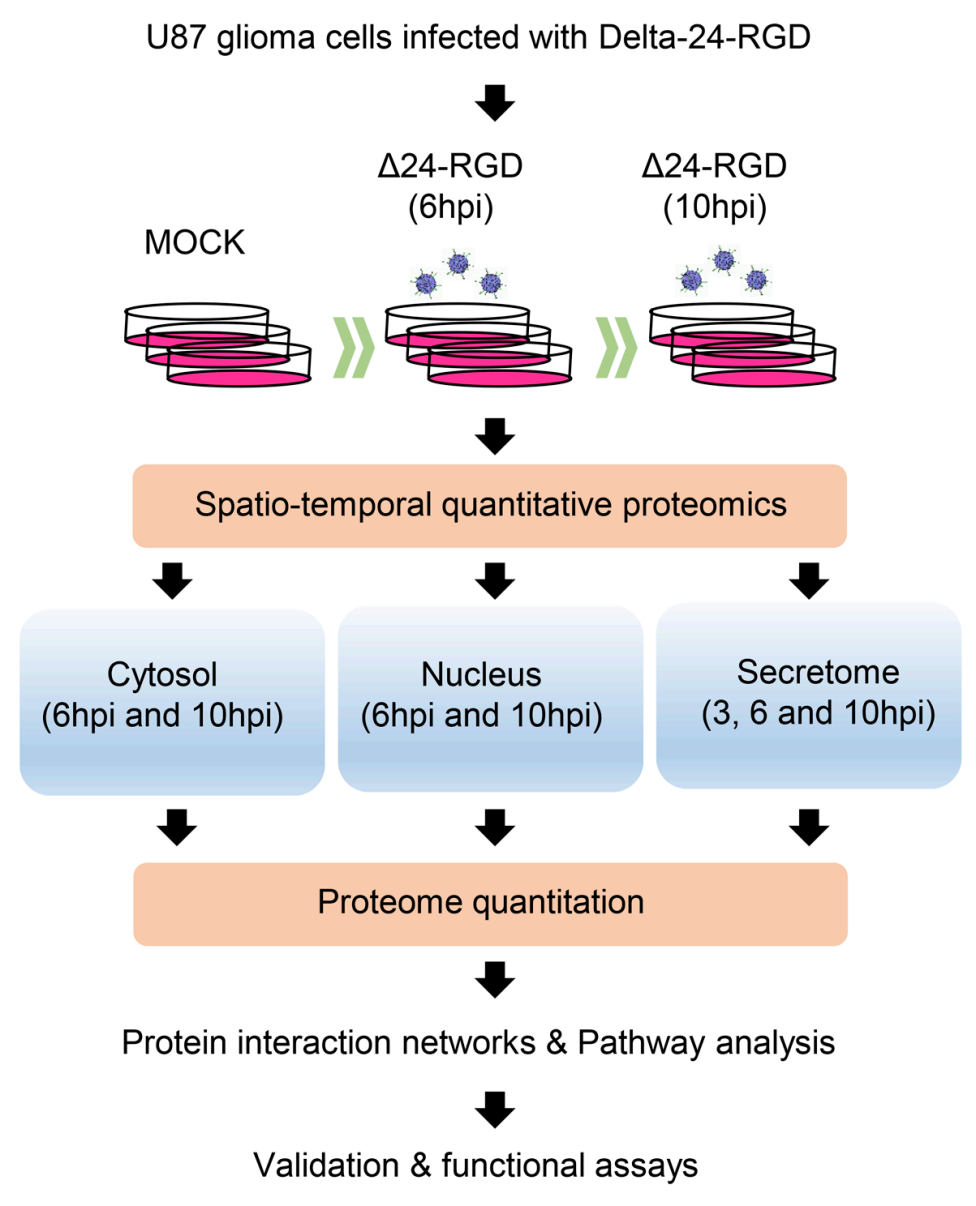
Hybrid proteomic approach to define spatial-temporal changes in organelle proteomes throughout Delta-24-RGD Infection

**Figure 2:**
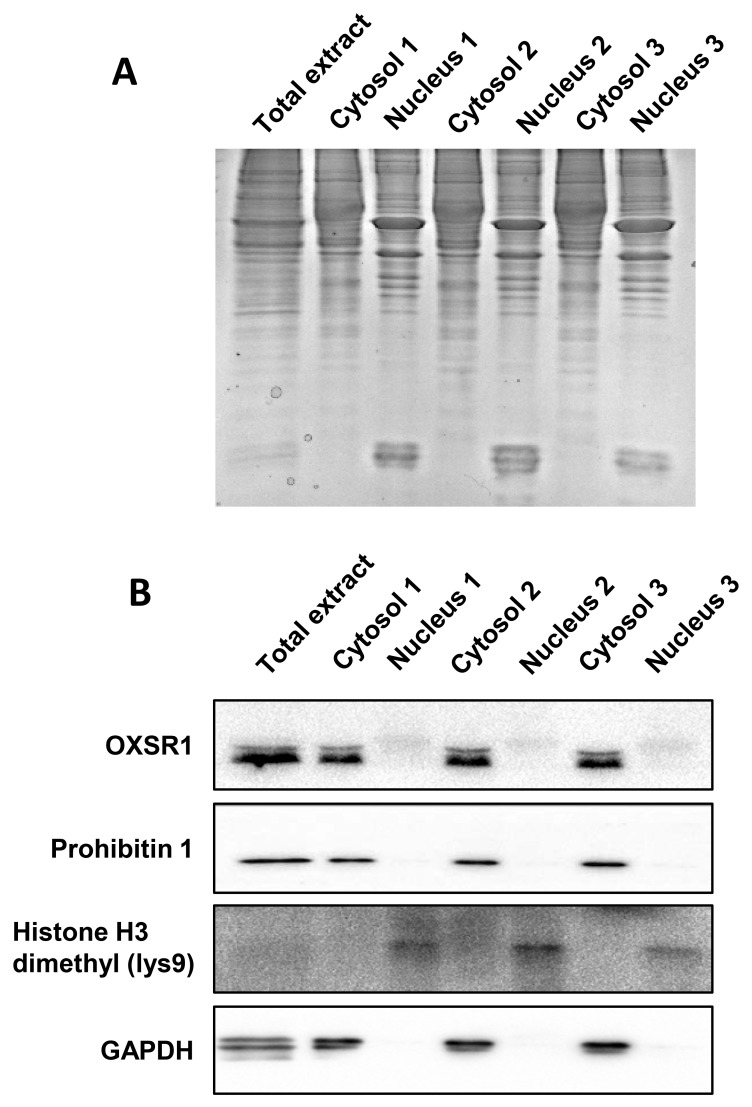
Validation of our enrichment procedure **(A)** Cytosolic and nuclear fractions resolved by SDS-PAGE electrophoresis. As shown in the upper panel, cytosolic and nuclear proteomes differ in the band profiles. **(B)** Specificity analysis by Western-blotting against specific cytosolic proteins (OXSR1, GAPDH), mitochondrial marker (Prohibitin-1) and nuclear histone (dimethyl-lysine 9 of histone H3).

**Figure 3:**
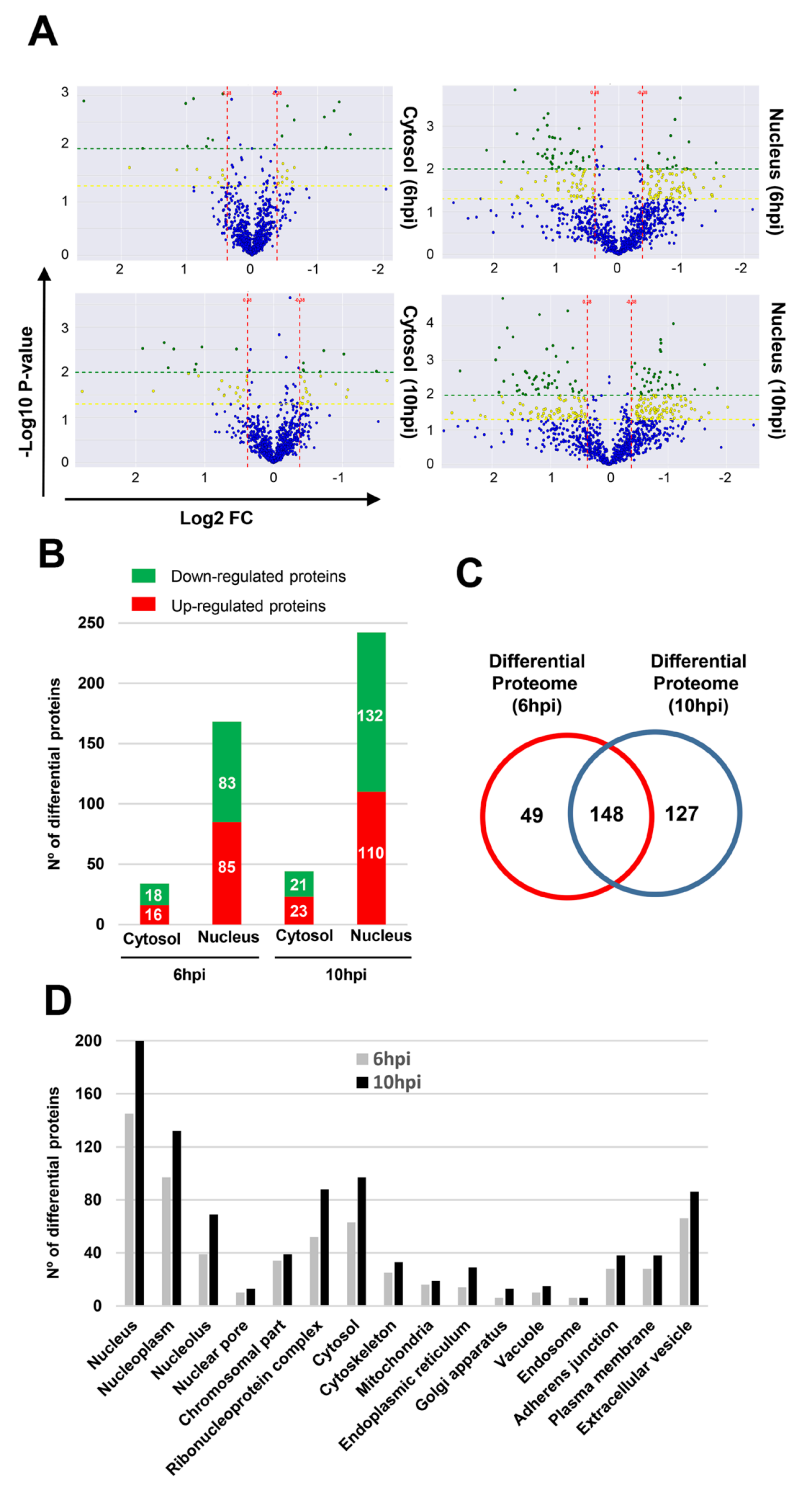
Differentially expressed proteins throughout early phases of Delta-24-RGD Infection **(A)** Volcano plots representing the fold-change of cytosolic and nuclear identified proteins with associated P values from the pair-wise quantitative comparisons of mock vs glioma-infected cells at 6 and 10hpi. In green, very significantly changed proteins (P < 0.01), in yellow, significantly changed proteins (P < 0.05) and in blue, unchanged cytosolic and nuclear proteins between the pair-wise comparisons. **(B)** Spatial-temporal differential proteome distribution at 6 and 10hpi. **(C)** Venn diagram of common and unique differential proteins between infection time-points. **(D)** Classification of glioma proteins affected by Delta-24-RGD based on subcellular localization using DAVID tool.

To analyze the early proteostasis imbalance induced by Delta-24-RGD, cytosolic and nuclear subcellular fractions were isolated from mock and U87-infected cells (6 and 10hpi). Two complementary proteome quantitation methods were used to monitor both protein localization and abundance within cytosol and nucleus during early phases of Delta-24-RGD infection (Figure 1). Detection of Serine/threonine-protein kinase OSR1, mitochondrial prohibitin, and GAPDH preferentially in the cytosolic fraction, in addition to major dimethylated histone H3 location in the nuclear fraction indicated the efficiency of the enrichment procedure (Figure 2). Among cytosolic and nuclear proteins consistently quantified during the time course (Figure 3A and 3B), 324 proteins tend to be differentially expressed between Mock and glioma-infected cells (Figure 3C, and Supplementary Tables 1 and 2). Proteome-wide exploration revealed that 202 protein products are differentially expressed at 6hpi, increasing the proteome alterations as the infection progresses (286 differential proteins at 10hpi) (Figure 3B, and Supplementary Figure 1). The most up-, and down-regulated proteins in both compartments are shown in Table 1. As shown in Figure 3B, minor cytosolic alterations were observed at 6 and 10hpi, whereas most changes in protein abundance were observed at nuclear level (Supplementary Figure 1). This was expected, as during adenovirus infection, there are severe structural and functional alterations in the nucleus of the host cell [24–26]. One of the most overexpressed protein in response to Delta-24-RGD infection is the Non-POU domain-containing octamer-binding protein (NONO). Modulation of NONO expression during Delta-24-RGD infection was verified by Western blotting in nuclear extracts and in total cell extracts (Supplementary Figure 2), confirming the increment of this transcriptional regulator during the infection, and partially validating the quantitative LC-MS/MS approach used in this study. Interestingly, subcellular distribution analysis of the glioma cell proteome modified by Delta-24-RGD also reveal an early alteration of protein components of adherents junctions and extracellular vesicles (Figure 3D).

**Table 1:** Top differentially expressed proteins during Delta-24RGD infection

	Nuclear proteins (6hpi)				Cytosolic proteins (6hpi)		
Gene name	Protein	Fold-change	p-value	Gene name	Protein	Fold-change	p-value
*F2*	Prothrombin	0.31	0.015	*HIST1H4A*	Histone H4	0.35	0.005
*SSRP1*	FACT complex subunit SSRP1	0.32	0.026	*H3F3B*	Histone H3 (Fragment)	0.40	0.001
*SUPT16H*	FACT complex subunit SPT16	0.34	0.007	*HIST1H1E*	Histone H1.4	0.42	0.002
*SCAF1*	Splicing factor, arginine/serine-rich 19	0.35	0.017	*HIST1H1B*	Histone H1.5	0.46	0.010
*MTA1*	Metastasis-associated protein MTA1	0.36	0.044	*HIST1H2BK*	Histone H2B type 1-K	0.47	0.003
*HIST1H1C*	Histone H1.2	0.36	0.040	*ERH*	Enhancer of rudimentary homolog	0.64	0.023
*HIST1H1B*	Histone H1.5	0.36	0.040	*SHMT2*	Serine hydroxymethyltransferase, mitochondrial	0.64	0.003
*H2AFY*	Core histone macro-H2A.1	0.39	0.027	*H2AFV*	Histone H2A.V	0.65	0.043
*RSL24D1*	Probable ribosome biogenesis protein RLP24	0.43	0.016	*SFPQ*	Splicing factor, proline- and glutamine-rich	0.69	0.002
*UBTF*	Nucleolar transcription factor 1	0.43	0.013	*SQRDL*	Sulfide:quinone oxidoreductase, mitochondrial	0.69	0.026
*RFC2*	Replication factor C subunit 2	2.73	0.012	*PSMD9*	26S proteasome non-ATPase regulatory subunit 9	1.33	0.038
*EIF3E*	Eukaryotic translation initiation factor 3 subunit E	2.87	0.007	*HDGF*	Hepatoma-derived growth factor	1.37	0.025
*EIF3L*	Eukaryotic translation initiation factor 3 subunit L	2.93	0.030	*TOMM70*	Mitochondrial import receptor subunit TOM70	1.43	0.043
*ARID2*	AT-rich interactive domain-containing protein 2	3.03	0.047	*SNX6*	Sorting nexin 6, isoform CRA_b	1.52	0.007
*EIF3H*	Eukaryotic translation initiation factor 3 subunit H	3.07	0.044	*A2M*	Alpha-2-macroglobulin	1.59	0.006
*RACK1*	Receptor of activated protein C kinase 1	3.08	0.002	*LTF*	Lactotransferrin	1.86	0.001
*G3BP1*	Ras GTPase-activating protein-binding protein 1	3.14	0.000	*NASP*	Nuclear autoantigenic sperm protein	3.18	0.010
*CPSF7*	Cleavage and polyadenylation-specificity factor subunit 7	3.54	0.007	*AHSG*	Alpha-2-HS-glycoprotein (Fetuin-A)	3.66	0.023
*SRSF2*	Serine/arginine-rich-splicing factor 2 (Fragment)	3.66	0.020				
*NONO*	Non-POU domain-containing octamer-binding protein	4.31	0.004				
*RRP7A*	Ribosomal RNA-processing protein 7 homolog A	0.25	0.022	*HIST1H2BK*	Histone H2B type 1-K	0.49	0.004
*HIST1H1C*	Histone H1.2	0.28	0.006	*HIST1H4A*	Histone H4	0.32	0.015
*F2*	Prothrombin	0.31	0.032	*H3F3B*	Histone H3 (Fragment)	0.35	0.010
*H2AFY*	Core histone macro-H2A.1	0.32	0.001	*HIST1H1E*	Histone H1.4	0.47	0.024
*SCAF1*	Splicing factor, arginine/serine-rich 19	0.34	0.043	*HIST1H1B*	Histone H1.5	0.48	0.036
*ARPC4*	Actin-related protein 2/3 complex subunit 4	0.36	0.026	*SHMT2*	Serine hydroxymethyltransferase, mitochondrial	0.53	0.018
*IMP3*	U3 small nucleolar ribonucleoprotein protein IMP3	0.36	0.007	*SERPINE1*	Plasminogen activator inhibitor 1	0.60	0.003
*NUP50*	Nuclear pore complex protein Nup50	0.38	0.025	*AKAP2*	A-kinase anchor protein 2	0.62	0.012
*UTP6*	U3 small nucleolar RNA-associated protein 6 homolog	0.38	0.016	*SUPT16H*	FACT complex subunit SPT16	0.63	0.010
*SSRP1*	FACT complex subunit SSRP1	0.39	0.049	*PCNA*	Proliferating cell nuclear antigen	0.69	0.032
*NONO*	Non-POU domain-containing octamer-binding protein	3.12	0.003	*RPL23*	60S ribosomal protein L23	1.31	0.015
*RRBP1*	Ribosome-binding protein 1	3.18	0.033	*DNAJC8*	DnaJ homolog subfamily C member 8	1.35	0.025
*NMT1*	Glycylpeptide N-tetradecanoyltransferase 1	3.51	0.016	*KYNU*	Kynureninase	1.40	0.035
*RACK1*	Receptor of activated protein C kinase 1	3.52	0.000	*A2M*	Alpha-2-macroglobulin	1.43	0.022
*ANXA2*	Annexin A2	3.56	0.011	*RPN2*	Dolichyl-diphosphooligosaccharide-prot. glycosyltransf. sub. 2	1.45	0.003
*RFC2*	Replication factor C subunit 2	3.69	0.008	*CNDP2*	Cytosolic non-specific dipeptidase	1.46	0.029
*ARID2*	AT-rich interactive domain-containing protein 2	4.33	0.042	*PSMD9*	26S proteasome non-ATPase regulatory subunit 9	1.55	0.021
*EIF3F*	Eukaryotic translation initiation factor 3 subunit F	4.58	0.038	*HDGF*	Hepatoma-derived growth factor	1.65	0.033
*PDCD6*	Programmed cell death protein 6	4.93	0.014	*LETM1*	LETM1 and EF-hand domain-containing protein 1	2.73	0.003
*RPS15*	40S ribosomal protein S15	6.43	0.032	*NASP*	Nuclear autoantigenic sperm protein	3.36	0.026

Differential proteins are ranked from most down-regulated to most up-regulated in both subcellular compartments at 6 and 10 hpi.

### Functional modules progressively disrupted in Delta-24-RGD-infected glioma cells

**Figure 4:**
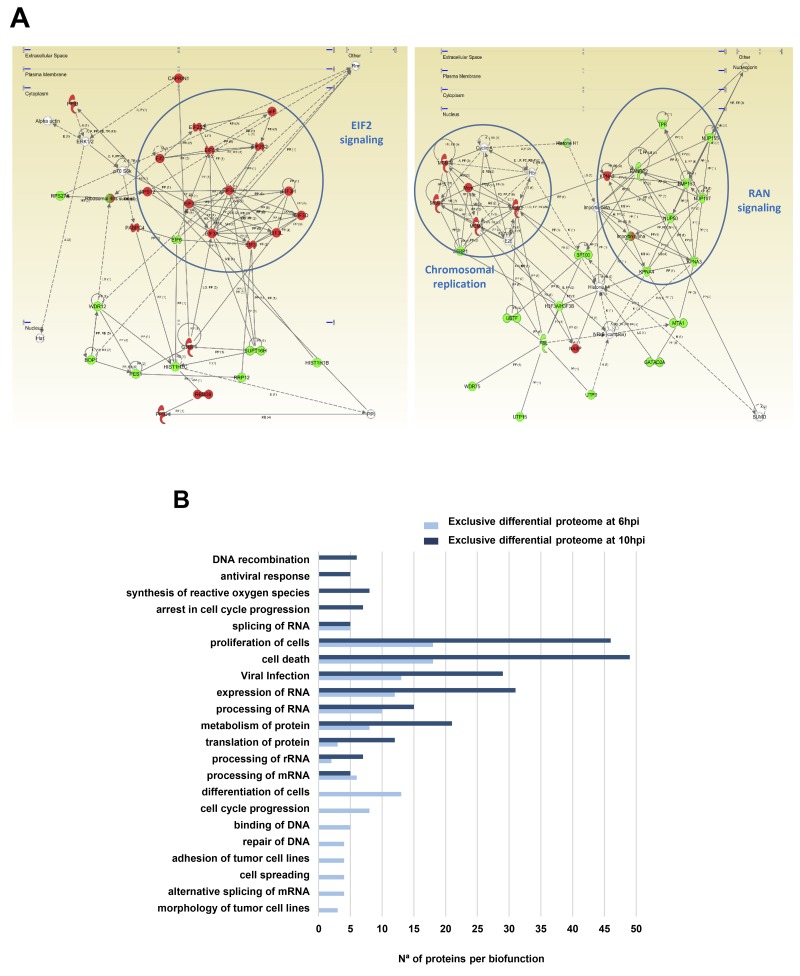
Functional metrics of the differential proteomic expression profile detected in glioma cells subjected to Delta-24-RGD infection **(A)** EIF2, MCMs, and RAN protein interactomes modulated by Delta-24-RGD in glioma cells. The up-regulation of protein intermediates involved in EIF2 signaling (left panel), down-regulation of proteins involved in RAN signaling (right panel), and over-expression of minichromosome maintenance (MCM) protein complex (right panel) are highlighted in both interactomes. The alteration in these pathways were detected at 6 and 10hpi. In green, proteins that were down-regulated; In red, proteins that were up-regulated in our data set. **(B)** Data-mining of the differential glioma proteome exclusively characterized at 6 or 10hpi. Biological processes and biofunctions modulated by Delta-24-RGD at 6 an 10hpi are shown.

To explore the cooperative action among differentially modulated proteins by Delta-24-RGD, we performed protein-scale interaction networks merging the cellular targets that tend to be de-regulated during infection. Protein interactome networks were constructed using IPA software. The integrative network-based workflow allowed to: i) decipher the molecular context of the cellular targets deregulated in each time point, ii) establish a framework to monitor potential interaction between deregulated targets and network modules during infection, and iii) to determine causal regulators of the time-dependent networks that may be considered as protein targets to modulate the infectivity process in glioma cells. Based on the altered common proteome between 6 and 10hpi (148 differentially expressed protein products) (Figure 3C), protein-interactome networks revealed that Delta-24-RGD mainly impacts on specific canonical pathways like RAN signaling (p-val: 2,08E-6), cell cycle control of chromosomal replication (p-val: 2,99E-6), and EIF2 signaling (p-val: 7,6E-10) (Figure 4A), indicating a progressive imbalance in the nucleo-cytoplasmic transport through the nuclear pore complex (NPC), and readjustments in DNA replication (p-val: 2,7E-8), transcription (p-val:7,7E-7), and mRNA translation (p-val: 4,1E-11) between 6 and 10hpi (Supplementary Figure 3). Moreover, protein clusters involved in cellular adhesion and differentiation was compromised at 6hpi (Figure 4B). In addition, our data pointed out a time-dependent deregulation of specific biological processes (Figure 4B). Protein clusters involved in RNA processing and proliferation/cell death pathways were mapped across both time points, while protein groups involved in cell cycle arrest, generation of ROS, and antiviral response were exclusively detected at 10hpi. (Figure 4B).

### Early temporal dynamics of STAT3, cJUN, NFκB and protein kinase C signaling pathways in glioma-infected cells

**Figure 5:**
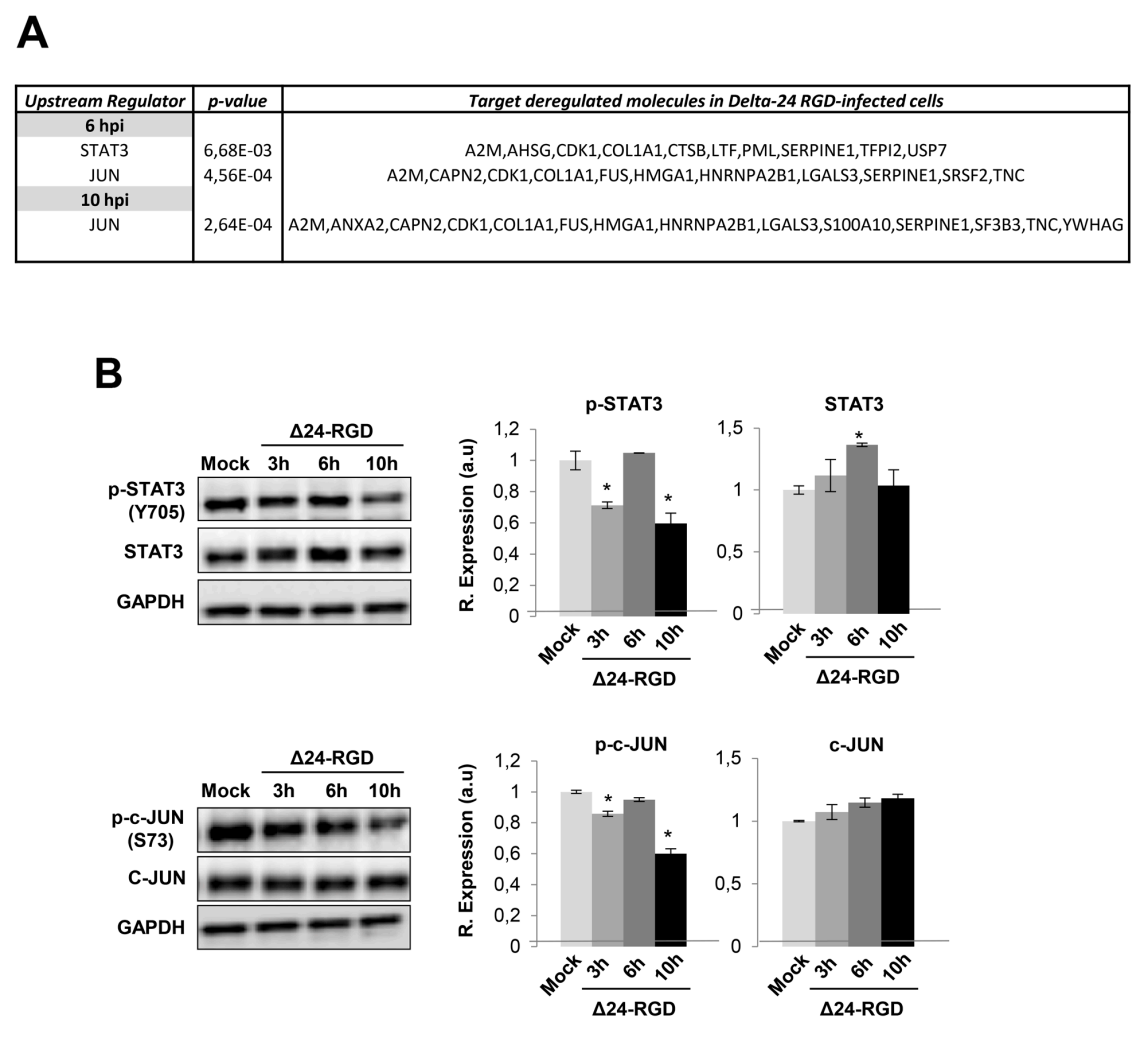
Activation profiling of STAT3 and c-JUN during Delta 24-RGD infection **(A)** Upstream regulators proposed by IPA software. **(B)** Levels and residue-specific phosphorylation of STAT3 and c-JUN at 3, 6, and 10hpi. Equal loading of the gels was assessed by Ponceau staining and hybridization with a GAPDH specific antibody. Representative Western blot images from three independent experiments are shown. Data are presented as mean ± SEM. ^*^P < 0.05 vs mock-infected cells.

**Figure 6:**
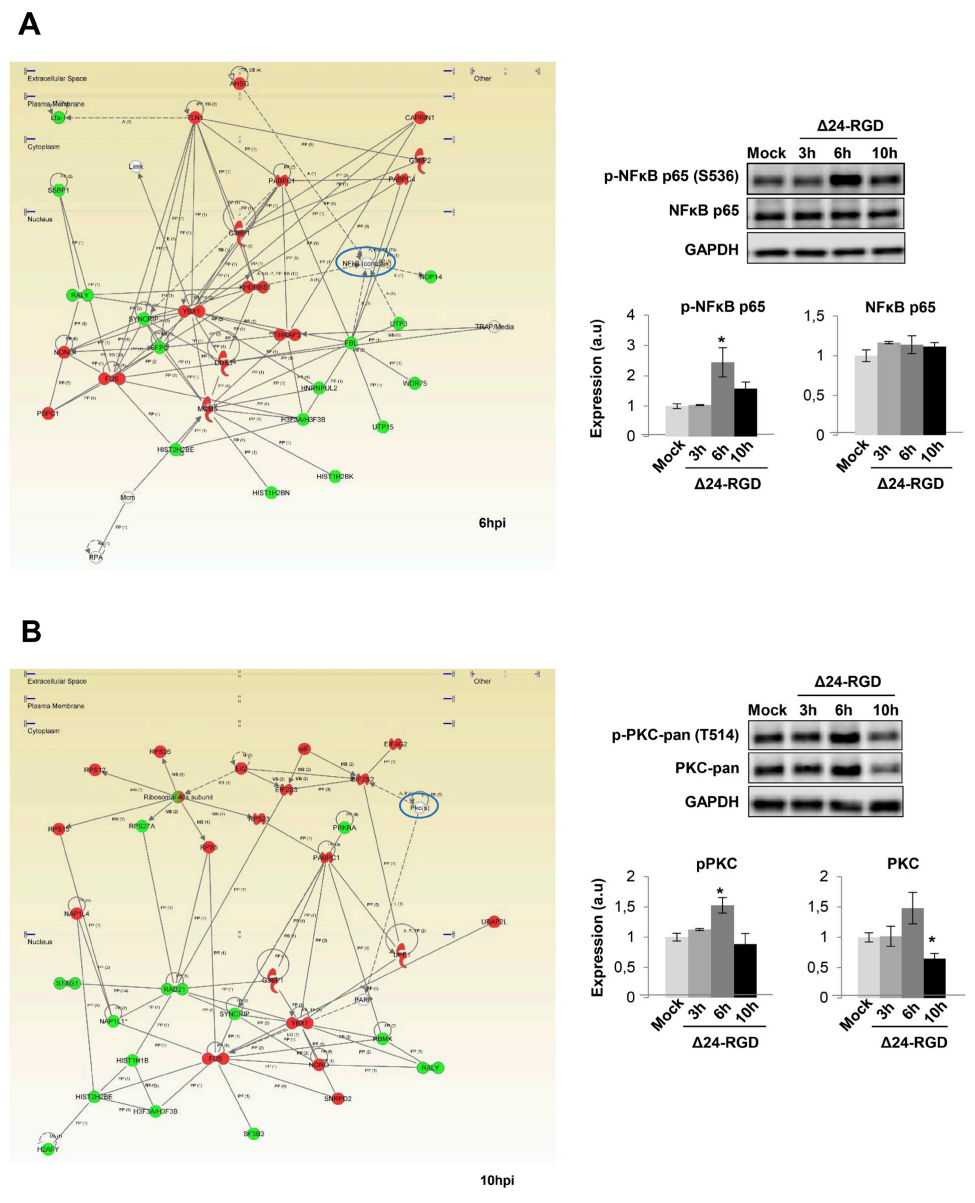
Delta 24-RGD modulates NFκB and PKC activity in early stages of infection **(A)** A cluster of NFkB target proteins displayed differentialexpression pattern at 6hpi, suggesting the impairment of this essential transcription factor. Activation of NFkB was furtherconfirmed by the increase of S536 phosphorylated levels at 6hpi. **(B)** PKC appeared as a main hub in the functional interactome derived from differential expressed proteome at 10hpi. A specific decrease in total PKC levels was evidenced by western-blotting at 10hpi. Equal loading of the gels was assessed by Ponceau staining and hybridization with a GAPDH specific antibody. Histograms of band densities derived from three independent experiments. Data are presented as mean ± SEM. ^*^P < 0.05 vs mock-infected condition. In green, proteins that were down-regulated; In red, proteins that were up-regulated in our data set.

We wanted to decipher which biological activities were occurring that may be responsible for protein expression changes induced by Delta-24-RGD. The predictive analysis of upstream modulation suggested potential impairment of upstream regulators such as STAT3, and JUN during the early stages of Delta-24-RGD (Figure 5A). Further experiments were performed to monitor the temporal activation profile of both transcription factors during the first ten hours of infection. As shown in Figure 5B, a transient inhibition of STAT3 and c-JUN was detected at 3 and 10hpi, maintaining normal activation levels at 6hpi. Moreover, signaling modulators like PKC, and NFκB appeared functionally interconnected in protein interactome networks modulated by Delta-24-RGD (Figure 6). Although changes in their expression were not detected in our proteome-wide analysis, the alteration of some of their targets may be compatible with a dysregulation of their functionality during early phases of Delta-24-RGD infection. As shown in Figure 6A, an increment in the phosphorylation of serine 536 of NFκB was specifically detected at 6hpi, suggesting a transient activation of NFκB. In addition, Western-blot using a specific antibody against phosphorylated PKC isoforms (at a residue homologous to activated Thr514 of human PKCγ) showed an increase in the activation state of PKC at 6hpi, probably due to a slight increment in total steady state levels, whereas total PKC levels significantly decreased at 10hpi (Figure 6B).

### Selectivity of Delta-24-RGD on the interference with specific survival routes

**Figure 7:**
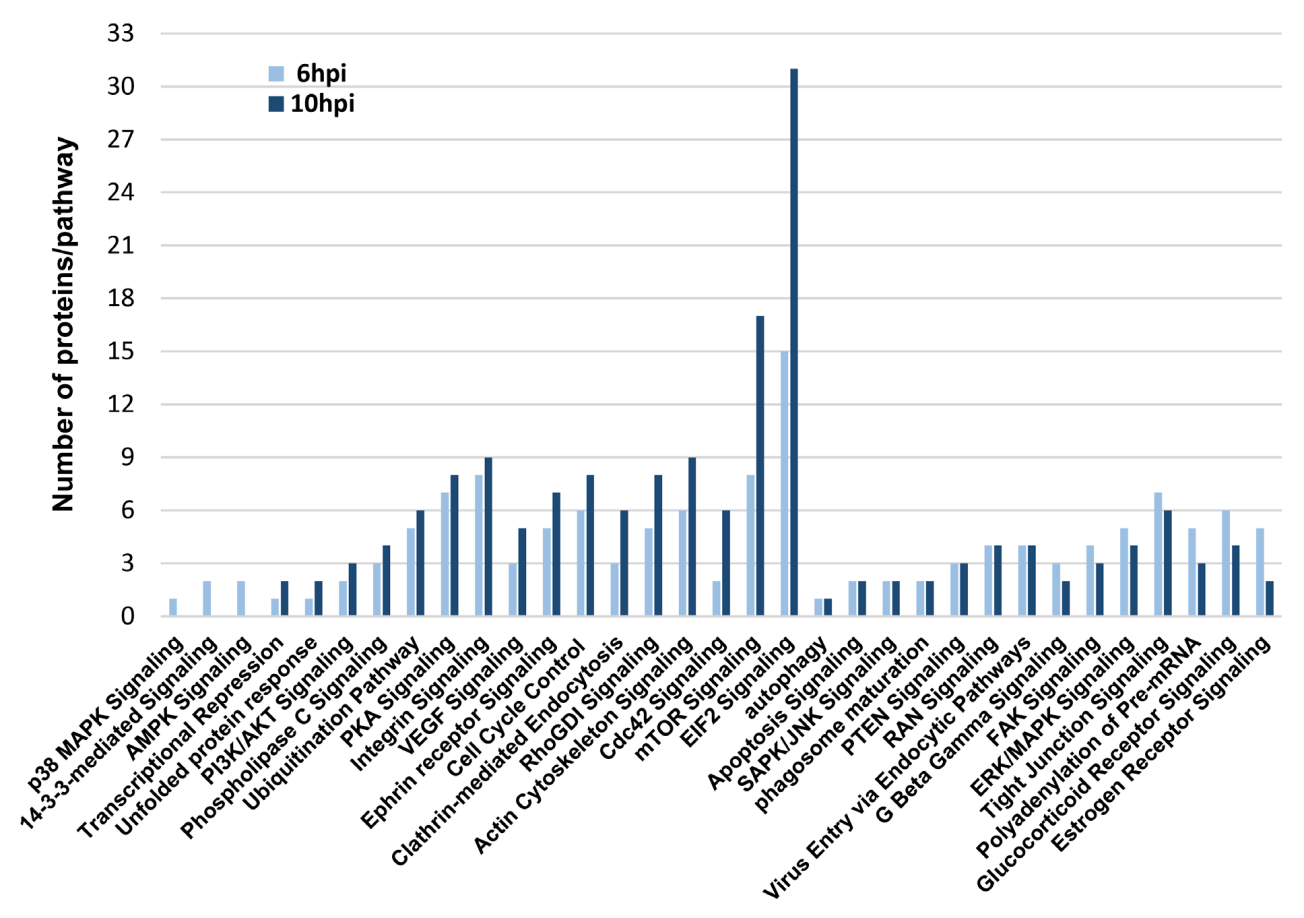
Protein clusters mapped into signaling pathways during Delta 24-RGD infection

**Figure 8:**
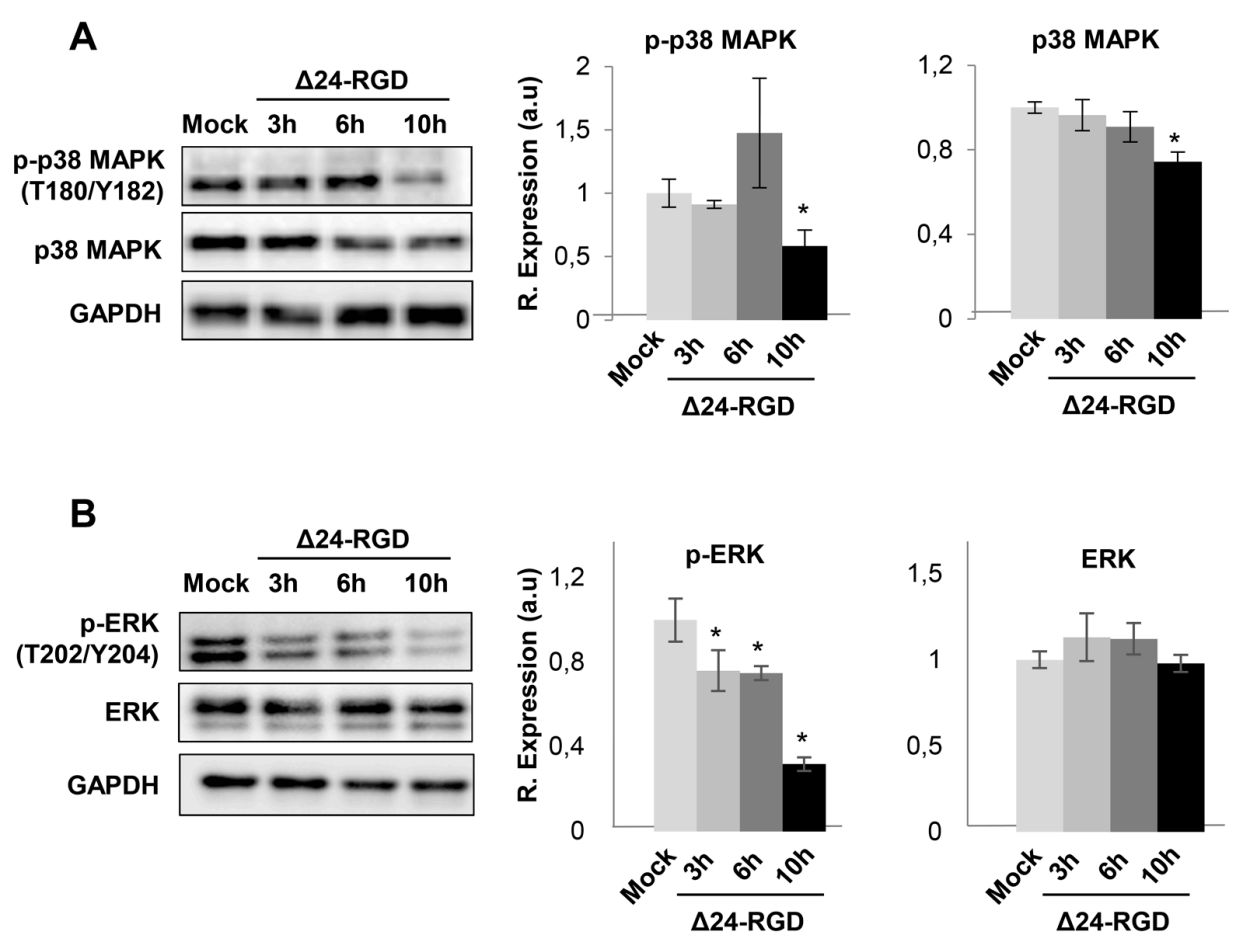
Signaling pathways disrupted in glioma cells upon Delta 24-RGD infection Levels and residue-specific phosphorylation of p38 MAPK **(A)**, and ERK1/2 **(B)**, in glioma-infected cells. Equal loading of the gels was assessed by Ponceau staining and hybridization with a GAPDH specific antibody. Right panels show histograms of band densities from three independent experiments. Data are presented as mean ± SEM. ^*^P < 0.05 vs mock-infected condition.

To deeply check the effects of Delta-24-RGD virus in the modulation of survival potential of glioma cells, a signaling pathway analysis was performed based on the glioma targets differentially expressed upon Delta-24-RGD infection. As shown in Figure 7, protein clusters involved in mTOR and EIF2 signaling were clearly mapped in both time points. The activation of both pathways has been previously characterized in adenoviral infections [27]. However, our bioinformatic workflow also predicted a plethora of signaling routes that may be potentially compromised during the first hours of infection. The activation status of specific survival pathways was monitored over time in infected-glioma cells. During the first 10hpi, no activation waves were detected in the kinase activity measurements of phospholipase C gamma (PLC-gamma), protein kinase B (AKT), stress-activated protein kinase/Jun-amino terminal kinase (SAPK/JNK), phosphoinositide-dependent protein kinase 1 (PDK1), and protein kinase A (PKA) (Supplementary Figure 4). However, p38MAPK activity was diminished at 10hpi, while the activation of ERK1/2 was compromised in a time-dependent manner (Figure 8). These data pointed out that Delta-24RGD specifically targets MAPK and p38 MAPK pathways during the first hours post-infection.

### Delta-24-RGD alters the glioma secretome: time-dependent production of specific cytokine subsets

**Figure 9:**
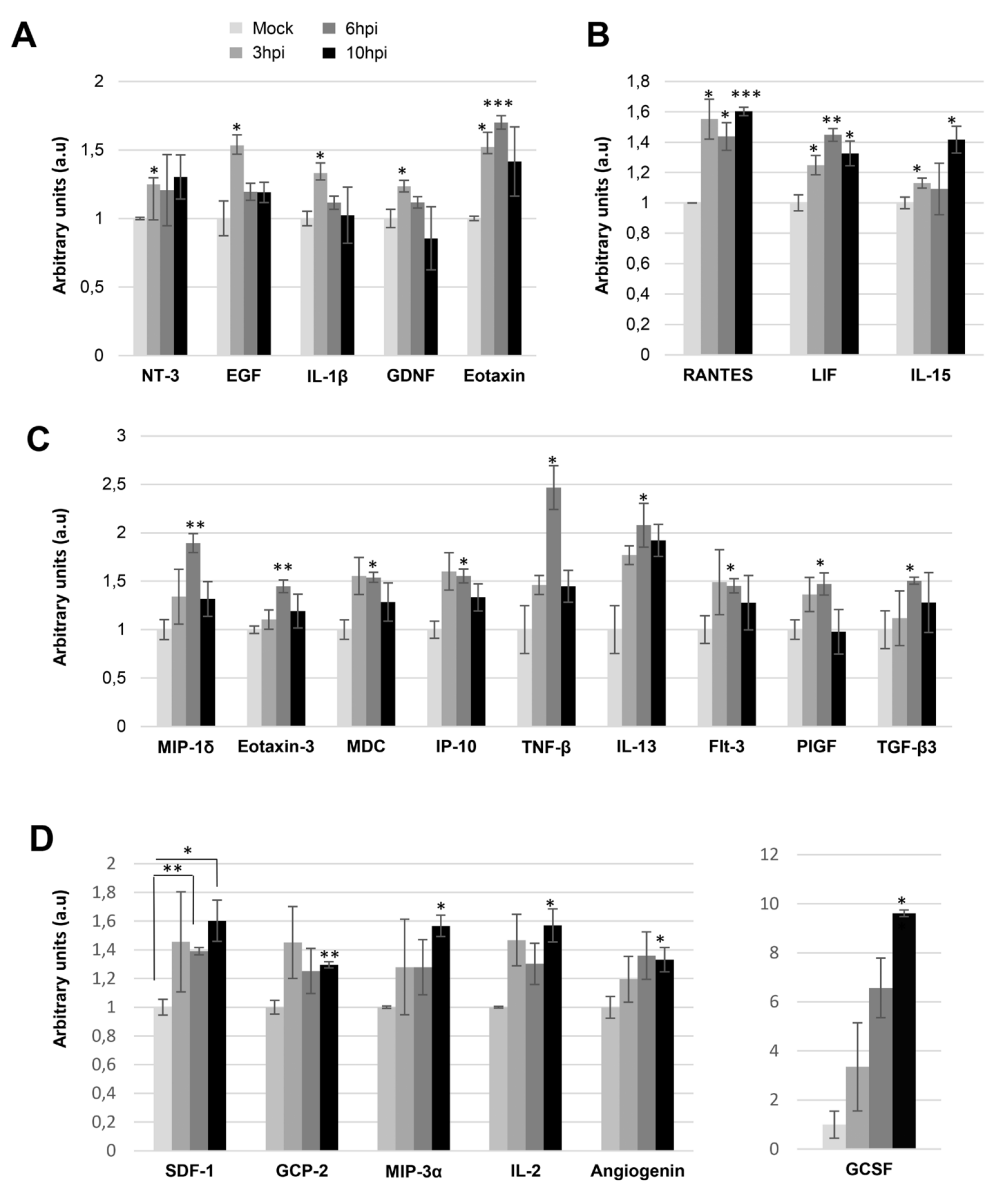
Delta 24-RGD induces early changes in the extracellular cytokine profiling of glioma cells A time-dependent analysis of 80 cytokines/growth factors was performed in the cell media of mock-infected glioma cells and glioma-infected cells (3, 6, and 10hpi) using a dot-blot protein array method. Three independent experiments were performed. Specific increments in cytokine production between 3 and 10 hpi are shown **(A-D)**. Data are presented as mean ± SEM. ^*^p < 0.05, ^**^p<0.01, and ^***^p<0.001 vs mock-infected condition.

In our attempt to decipher the early metabolic response of glioma cells during the Delta-24-RGD infection, we have performed a complementary secretome analysis of glioma cells during Delta-24-RGD infection. Considering that the monitorization of cytokines and growth factors secreted by glioma cells to the tumor microenvironment may provide new insights into the early modulation of the immune response induced by the oncolytic Delta-24-RGD vector, circulating inflammatory cytokines and growth factors were analyzed in the cell media of Delta-24-RGD-infected cells. Among the 80 secreted cell–cell signaling molecules analyzed (Supplementary Figure 6), 23 were significantly increased in the secretome of infected-glioma cells in a time-dependent manner (Figure 9). Cytokines and growth factors like neurotrophin-3 (NT-3), endothelial growth factor (EGF), IL-1beta, and glial cell-derived neurotrophic factor (GDNF) were specifically increased at 3hpi, while eotaxin was overproduced at 3 and 6hpi (Figure 9A). Moreover, the increment in the secretion of Rantes (CCL5), leukemia inhibitor factor (LIF), and IL-15 was evidenced between 3 and 10hpi (Figure 9B). Chemotactic factors like MIP-1delta (CCL15), eotaxin-3, MDC (CCL22), and IP-10, together with TNF-beta, TGF-beta3, Flt3 (CD135), and multitasking cytokines such as IL13 and Placental growth factor (PlGF) were upregulated in the secretome from Delta-24-RGD-infected cells at 6hpi (Figure 9C). At 10hpi, an increment in the levels of a panel of pleiotropic cytokines (SDF1, GCP-2, MIP-3 alpha, GCSF, IL-2, and angiogenin) was also significantly detected (Figure 9D). Interestingly, these data pointed out that Delta-24-RGD triggers a dynamic production of pleiotropic factors to the extracellular environment, suggesting that these secreted molecules may regulate the glioma growth, and differentiation states together with the inflammatory cell recruitment and activation *in vivo*. To explore the cooperative action among differentially intracellular and extracellular molecules induced by Delta-24-RGD, we have performed additional pathway analysis merging the proteomic datasets and the secretome information. As shown in Table 2, several common altered pathways were detected at both time points, although differential protein intermediates were different. Most of them are related with cell communication, immune system, and infection response.

**Table 2:** Pathway mapping of intracellular deregulated proteins and the differential secretome

PATHWAY	PROTEINS (6hpi)	PROTEINS (10hpi)
**Dendritic Cell Maturation**	**LTA**, COL1A1	**IL15**, COL1A1
**CXCR4 Signaling**	**CXCL12**, RACK1, GNB2	MYL6, **CXCL12**, RACK1
**CCR5 Signaling in Macrophages**	**CCL5**, RACK1, GNB2	**CCL5**, RACK1
**ERK5 Signaling**	**LIF**, YWHAB	YWHAG**, LIF**
**Glucocorticoid Receptor Signaling**	A2M, TAF15**, TGFB3**, HSPA1A/HSPA1B, **CCL5**, ARID2, SERPINE1, **CCL11, IL13**, SMARCC2, KRT9, KRT14	A2M**, IL2, CCL5**, ARID2, SERPINE1, HSP90AB1, KRT14
**Axonal Guidance Signaling**	**CXCL12**, CDC42, RACK1, ITGA3, RAC2, GNB2	MYL6, **CXCL12**, CDC42, ACTR2, RACK1, ITGA3, ARPC5L, RAC2, ARPC1A
**Crosstalk between Dendritic Cells and Natural Killer Cells**	TLN1, ACTG1, **LTA**	**IL2, IL15**, ACTG1
**Leukocyte Extravasation Signaling**	**CXCL12**, CDC42, ACTG1, ITGA3, RAC2	MYL6, **CXCL12**, CDC42, ACTG1, ITGA3, RAC2
**Granulocyte Adhesion and Diapedesis**	**CXCL12, CCL26, CCL22, CCL5**, ITGA3, **CXCL10, CCL15, CCL11**	**CXCL6, CXCL12, CCL5**, ITGA3, **CCL20**, CSF3
**Agranulocyte Adhesion and Diapedesis**	**CXCL12, CCL26, CCL22**, ACTG1**, CCL5**, ITGA3**, CXCL10, CCL15, CCL11**	**CXCL6**, MYL6, **CXCL12**, ACTG1**, CCL5**, ITGA3, **CCL20**
**Ephrin B Signaling**	**CXCL12**, CDC42, RACK1, RAC2, GNB2	**CXCL12,** CDC42, RACK1, RAC2
**Ephrin Receptor Signaling**	**CXCL12**, CDC42, RACK1, ITGA3, RAC2, GNB2	**CXCL12**, CDC42, ACTR2, RACK1, ITGA3, ARPC5L, RAC2, ARPC1A
**Antiproliferative Role of TOB in T Cell Signaling**	**TGFB3**, PABPC1, PABPC4	**IL2**, PABPC1, PABPC4
**HMGB1 Signaling**	**TGFB3**, CDC42**, LTA, LIF**, SERPINE1**, IL13**	**IL2**, CDC42, **LIF**, SERPINE1
**Wnt/β-catenin Signaling**	**TGFB3**, RPS27A	
**Aryl Hydrocarbon Receptor Signaling**	**TGFB3**, MCM7	
**p38 MAPK Signaling**	**TGFB3**, H3F3A/H3F3B	
**Fc Epsilon RI Signaling**	**IL13**, RAC2	
**Cell Cycle: G1/S Checkpoint Regulation**	**TGFB3**, PA2G4	
**Pancreatic Adenocarcinoma Signaling**	**TGFB3**, CDC42, PA2G4	
**RAR Activation**	**TGFB3**, PML, ARID2, SMARCC2	
**TGF-β Signaling**	**TGFB3**, CDC42, SERPINE1	
**Cyclins and Cell Cycle Regulation**	**TGFB3**, PA2G4, CDK1	
**CCR3 Signaling in Eosinophils**	**CCL26**, RACK1, **CCL11**, GNB2	
**Protein Kinase A Signaling**	HIST1H1C**, TGFB3**, RACK1, H3F3A/H3F3B, HIST1H1E, HIST1H1B, YWHAB, GNB2	
**Tight Junction Signaling**	CSTF3**, TGFB3**, CDC42, CSTF2, ACTG1, CPSF1, CPSF2, NUDT21	
**PKCθ Signaling in T Lymphocytes**		**IL2**, RAC2
**Telomerase Signaling**		**IL2**, HSP90AB1
**CD28 Signaling in T Helper Cells**		**IL2**, CDC42, ACTR2, ARPC5L, ARPC1A
**Recognition of Bacteria and Viruses**		EIF2S1, **IL2, CCL5, LIF**

Cytokines and growth factors deregulated at extracellular level during Delta-24RGD infection were jointly analyzed with intracellular deregulated proteins. Pathways represented at 6 and 10hpi by IPA software are shown.

## DISCUSSION

We consider that a quantitative knowledge of the intracellular and extracellular glioma proteomes may help to understand the early effects that occur during Delta-24-RGD infection, providing potential therapeutic targets that may enhance the efficacy of the adenoviral therapy. One of our goals was to generate extensive data on the functional groups of glioma proteins early deregulated during Delta-24-RGD infection. For that, we have applied a system biology approach performing different molecular networks and protein profiling analysis to identify biologically relevant pathways from large-scale glioma proteome data. From a functional point of view, specific proteomic fingerprints were dynamically modulated in a time-dependent manner early in infection. Interestingly, our results partially overlapped to those of a recent proteogenomic analysis in cervical tumor cells infected with wild-type adenovirus serotype 5 (Ad5) in similar infectivity conditions [28], partially validating our proteomic approach (Supplementary Figure 5), and typifying common molecular mechanisms associated to the infection of tumor cells with an Ad5 infection. STAT3 is a critical mediator of tumorigenesis, and tumor progression in glioblastoma [29, 30]. Interestingly, Delta-24-RGD inhibits the activity of STAT3 at 10hpi. Previous studies have demonstrated that the specific blocking of STAT3 produced by oncolytic adenoviral mutants induces a potent antitumoral activity in different tumoral backgrounds [31, 32]. In line with these findings, the STAT3 inhibition may positively contribute to the antitumoral effect induced by Delta-24-RGD. The activation of NF-κB has been associated with resistance to different cell death strategies in GBM, and NF-κB-target genes (citokines, cell-cycle regulators, and anti-apoptotic proteins between others) have been proposed to influence the invasion capacity and resistance to chemotherapy [33]. Furthermore, previous studies point out that adenovirus infection induces the activation of NF-κB in non-tumoral cells [34–36], and also in cancer cell lines [37, 38]. It has been shown that inhibition of NF-κB enhanced the cytotoxicity of oncolytic adenovirus in ovarian and colorectal carcinoma cell lines [37], whereas some replication-competent adenoviral vectors strongly reduced NF-κB activity, enhancing apoptosis in esophageal cancer cells [39]. Therefore, it may be hypothesized that the potential transient NF-κB activation observed in Delta-24-RGD-infected glioma cells may be explained not only by the infection itself, but also as a protection mechanism induced by glioma cells to counteract the deleterious effects on glioma metabolism caused by Delta-24-RGD infection. It is important to note that the activation profiling of NF-κB protein clusters is extremely complex, being highly dependent on the biological context/stimuli [40], and many post-translational modifications (PTMs) have been characterized (see http://www.uniprot.org/uniprot/Q04206), being still unclear how the tangled crosstalk between all PTMs regulates the ability of NF-κB proteins to induce or to repress defined target genes upon adenoviral infections. c-Jun accumulation is robustly elevated in human glial tumors and in glioblastoma cell lines, contributing to the malignant properties of the cells [41]. Its transcriptional activity is regulated by phosphorylation at Ser63 and Ser73 [42]. Interestingly, an increased in c-Jun phosphorylation (Ser63) has been previously detected in Delta-24-RGD-infected glioma cells late in infection (24hpi) [23]. Although this effect was observed using a higher viral load (MOI=50), the early c-Jun inhibition demonstrated by the drop of phospho-c-Jun (Ser73) levels at 3 and 10hpi, suggest that c-Jun phosphoproteome is highly dynamic during the adenoviral life cycle, maintaining a high activation state at advanced stages, needed for the adenoviral-mediated autophagic process [23]. Several pathways have been monitored during Delta-24-RGD infections [23]. Although Akt is not induced early in Delta-24-RGD infection, it has been demonstrated that the adenoviral vector increases Akt phosphorylation in U87 MG glioma cells at 24-48hpi [23]. Moreover, no significant changes in ERK1/2 phosphorylation levels were detected in Delta-24-RGD-infected lung fibroblasts (16-48hpi) [23]. However, Delta-24-RGD induces a time-dependent inactivation of ERK1/2 and a decrease in total and phospho-p38 levels during the first hours post-infection in glioma cells. It has been clearly demonstrated that adenovirus vectors induce an early ERK1/2 and p38 activation during cell entry in nontransformed kidney cells, although the activation of ERK may differ between cell types [43]. Moreover, the constitutively activated ERK is one of the key tumorigenic effectors present in the majority of glioblastoma variants [44], and its signaling is involved in migration and invasion [45]. In fact, blocking ERK activity reduces glioma tumorigenicity, and even sensitizes to chemotherapy [46, 47], suggesting that Delta-24-RGD-induced ERK inactivation may be a positive molecular event in the synergistic anti-glioma effects previously observed in the combination of Delta-24-RGD with chemotherapy [14–16]. Like ERK, p38 MAPK is upregulated and activated in GBM, contributing to tumor invasion and metastasis [48, 49]. Its inhibition sensitizes GBM cells to cytotoxic therapy and enhances the immune response [48, 50]. Therefore, it may be hypothesized that Delta-24-RGD compromises the glioma cell survival potential through the attenuation of ERK1/2 and p38 MAPK signaling. In contrast, an increment in phosphorylated PKC isoforms was detected early in Delta-24-RGD infection. Interestingly, PKC has been shown to play a critical role in the cellular entry of viruses [51]. Although PKC activity has been related to the growth regulation of malignant gliomas [52, 53], our data suggest that PKC activation may be a viral-induced signal to guarantee the adenoviral early gene expression [51]. However, the tangled regulatory mechanisms that govern the PKC signaling needs further exploration, in order to elucidate the specific role of each PKC isoform during initial stages of Delta-24-RGD infection.

It has been recently proposed that the immune system plays a pivotal role in the therapeutic efficacy of oncolytic Delta-24-RGD therapy of glioma [54, 55]. The induction of the chemokine IP-10 through capsid dependent activation of NF-κB by recombinant adenoviruses, represents an important early step in the development of host immunity against these vectors [43, 56]. Delta-24-RGD tends to induce the expression of IP-10, together with macrophage inflammatory proteins (MIPs), and IL-1beta in brains derived from mice bearing intracranial glioma tumors [54]. This induction has been related to the attraction of macrophages, CD4+ and CD8+ T-cells to the tumors [12, 54]. In line with these findings, an overproduction of IP-10, IL-1beta, MIP-1 delta and -3 alpha was also detected in the secretome of glioma-infected cells. Interestingly, a panel of other chemoattractant factors and multitasking cytokines has been unveiled in our study in response to Delta-24-RGD treatment. Therefore, it may be hypothesized that the secretion of cytokines like Rantes, PIGF, SDF1, IL-2 and IL-15 by Delta-24-RGD-infected glioma cells might shed new light to the proliferation and influx of immune cells recently observed in glioma tumors treated with Delta-24-RGD-based oncolytic adenovirus [57].

## MATERIALS AND METHODS

### Materials

The following reagents and materials were used: anti-GAPDH (Calbiochem), Anti-OXSR1, anti-NONO (Abcam), anti-Prohibitin-1 (cell signaling), anti-dimethylated histone H3 (Lys9) (Upstate), anti-p38 MAP kinase, anti-phospho p38 MAP kinase (Thr180/Tyr 182), anti-PDK1, anti-phospho PDK1 (S241), anti-PKC-Pan, anti-phospho PKC-pan (T514), anti-pAkt (Ser473), anti-Akt, anti-pERK1/2 (Thr202/Tyr204), anti-ERK1/2, anti-STAT3, anti-phospho STAT3 (Y705), anti-cJUN, anti-phospho cJUN (S73), anti-NF-κB p65, anti-NF-κB phospho-p65 (S536), anti-PLCgamma, anti-phospho PLCgamma (Y783), anti-SAPK/JNK, and anti-phospho SAPK/JNK (T183/Y185) (Cell Signaling). Electrophoresis reagents were purchased from Bio-rad and trypsin from Promega.

### Virus production, culture and treatment of malignant glioma cells

The construction of Delta-24-RGD has been previously described [8, 9]. U87 MG glioma cells (ATCC: HTB-14) were cultured in DMEM/F12-GlutaMAX (Gibco 10565018) supplemented with 10% FBS, and 1% penicillin/streptomycin. 3.5x10^6^ U87 MG cells were infected with Delta-24-RGD at multiplicity of infection (MOI) of 25. After incubation for 30 minutes with DMEM/F12 1% penicillin/streptomycin at 37 °C, the double of the volume of DMEM/F12-GlutaMAX (Gibco 10565018) supplemented with 10% FBS and 1% penicillin and streptomycin was added to the previous media. Cells were incubated under the same conditions during the indicated periods of time.

### Subcellular fractionation

After the indicated periods of time, the media was removed and the cells were washed with 1X cold PBS. Then, 500μl of NP40 buffer with protease inhibitors was added and cells were harvested. After a centrifugation step (1 minute, 18000xg, at 4°C), the supernatant was collected in a new eppendorf (cytosolic fraction). The pellet was washed with PBS and centrifuged for 1 minute (1 minute, 18000xg, at 4°C). The supernatant was discarded. The pellet (nuclear fraction) was resuspended in 50μl of lysis buffer (7M urea, 2M thiourea, 50mM DTT) and let on ice for 30 minutes, spinning and vortexing each 10 minutes. After a sonication step, the lysate was centrifuged for 20 minutes at 20000xg at 15°C. The supernatant was transfer to a new eppendorf and the pellet discarded. Protein concentration of both subcellular fractions was measured with the Bradford assay kit (Bio-rad).

### Cytosolic proteome analysis

A shotgun comparative proteomic analysis of cytosolic fractions using iTRAQ (isobaric Tags for Relative and Absolute Quantitation) was performed [58]. Global experiments were carried out with two/three biological replicates in each experimental condition. Cytosolic extracts (300 μg) were precipitated with methanol/chloroform, and pellets dissolved in 0.5M triethylammonium bicarbonate (TEAB), 6M urea. Protein quantitation was performed with the Bradford assay kit (Bio-Rad). iTRAQ labeling of each sample was performed according to the manufacturer’s protocol (Sciex). Briefly, a total of 80 μg of protein from each cellular condition was reduced with 50 mM tris (2-carboxyethyl) phosphine (TCEP) at room temperature for 1 h, and cysteine residues were alkylated with 200 mM methylmethanethiosulfonate (MMTS) at room temperature for 10 min. Protein enzymatic cleavage was carried out with trypsin (Promega; 1:50, w/w) at 37 °C for 16 h. Each tryptic digest was labelled according to the manufacturer’s instructions with one isobaric amine-reactive tags as follows: Tag113, Mock-infected U87 cells-1; Tag114, Mock-infected U87 cells-2; Tag115, U87-infected cells (6hpi)-1; Tag116, U87-infected cells (6hpi)-2; Tag117, U87-infected cells (6hpi)-3;Tag118, U87-infected cells (10hpi)-1; Tag119, U87-infected cells (10hpi)-2; Tag121, U87-infected cells (10hpi)-3. After 2h incubation, the set of labelled samples were pooled and evaporated in a vacuum centrifuge. To increase the proteome coverage, the peptide pool was fractionated by SCX chromatography. Briefly the sample was first dissolved in 10mM KH2PO4, 20% ACN, pH:3, sonicated and centrifuged for 3 minutes at 18000xg at RT. The pellet was discarded and the pH ˂ 3 was adjusted with formic acid (FA). Peptides were eluted with KCl at an increasing gradient from 1mM to 500mM. 8 different fractions were collected. Purification and concentration of peptides was performed using C18 Zip Tip Solid Phase Extraction (Millipore). Then the sample was evaporated under vacuum and reconstituted into 10μl of 2% acetonitrile, 0.5% FA, 98% MilliQ-H20 prior to mass spectrometric analysis.

### Nuclear proteome analysis

Nuclear extracts from Mock-infected, and U87-infected cells (at 6 and 10hpi) were diluted in Laemmli sample buffer and loaded into a 1 mm thick polyacrylamide gel with a 4% stacking gel casted over a 12.5% resolving gel. The run was stopped as soon as the front entered 3 mm into the resolving gel so that the whole proteome became concentrated in the stacking/resolving gel interface. Bands were stained with Coomassie Brilliant Blue and excised from the gel. Protein enzymatic cleavage (15ug) was carried out with trypsin (Promega; 1:20, w/w) at 37°C for 16 h as previously described [59]. Purification and concentration of peptides was performed using C18 Zip Tip Solid Phase Extraction (Millipore).

### Mass spectrometry

Peptides mixtures were separated by reverse phase chromatography using an Eksigent nanoLC ultra 2D pump fitted with a 75 μm ID column (Eksigent 0.075 x 250 mm). Samples were first loaded for desalting and concentration into a 0.5 cm length 100 μm ID pre-column packed with the same chemistry as the separating column. Mobile phases were 100% water 0.1% formic acid (FA) (buffer A) and 100% Acetonitrile 0.1% FA (buffer B). For cytosolic peptide mixtures (iTRAQ approach), the Column gradient was developed in a 135 min two step gradient from 5% B to 25% B in 120 min and 25%B to 70% B in 15 min. Column was equilibrated in 95% B for 9 min and 5% B for 14 min. In the case of nuclear peptide mixtures (label-free approach), the column gradient was developed in a 240 min two step gradient from 5% B to 25% B in 210 min and 25%B to 40% B in 30 min. Column was equilibrated in 95% B for 9 min and 5% B for 14 min. During all processes, precolumn was in line with column and flow maintained all along the gradient at 300 nl/min. Eluting peptides from the column were analyzed using a Sciex 5600 Triple-TOF system. Information data acquisition was acquired upon a survey scan performed in a mass range from 350 m/z up to 1250 m/z in a scan time of 250 ms. Top 25-35 peaks were selected for fragmentation. For cytosolic peptides mixtures: the minimum accumulation time for MS/MS was set to 75 ms giving a total cycle time of 2.1 s. Product ions were scanned in a mass range from 100 m/z up to 1500 m/z and excluded for further fragmentation during 15 s. In the case of nuclear peptides mixtures: Minimum accumulation time for MS/MS was set to 100 ms giving a total cycle time of 3.8 s. Product ions were scanned in a mass range from 230 m/z up to 1500 m/z and excluded for further fragmentation during 15 s. The MS/MS data acquisition was performed using Analyst 1.7.1 (Sciex) and spectra files were processed through Protein Pilot Software (v.5.0.1-Sciex) using Paragon™ algorithm (v.5.0.1) for database search [60], Progroup™ for data grouping, and searched against the concatenated target-decoy UniProt proteome reference database (Human database Proteome ID: UP000005640, 70902 proteins, December 2015 plus adenovirus HAv5 database UP000004992, 31 proteins, September 2016). False discovery rate was performed using a non lineal fitting method [61] and displayed results were those reporting a 1% Global false discovery rate or better. The mass spectrometry proteomics data have been deposited to the ProteomeXchange Consortium (http://proteomecentral.proteomexchange.org) [62] via the PRIDE partner repository with the data set identifiers PXD008023 (nuclear data) and PXD008022 (cytosolic data).

### Data analysis for cytosolic proteomes

Relative quantification and protein identification were performed with the ProteinPilot™ software (version 5.0; Sciex) using the Paragon™ algorithm as the search engine. The search parameters allowed for cysteine modification by MMTS and biological modifications programm in the algorithm (i.e. phosphorylations, amidations, semitryptic fragments, etc.). Reporter ion intensities were bias corrected for the overlapping isotope contributions from the iTRAQ tags according to the certificate of analysis provided by the reagent manufacturer (Sciex). The peptide and protein selection criteria for relative quantitation were performed as follows. Only peptides unique for a given protein were considered for relative quantitation, excluding those common to other isoforms or proteins of the same family. Proteins were identified on the basis of having at least one peptide with an ion score above 99% confidence. Among the identified peptides, some of them were excluded from the quantitative analysis for one of the following reasons: (i) The peaks corresponding to the iTRAQ labels were not detected; (ii) the peptides were identified with low identification confidence (<1.0%); (iii) the sum of the signal-to-noise ratio for all of the peak pairs was <6 for the peptide ratios. The protein sequence coverage (95% conf.) was estimated for specific proteins by the percentage of matching amino acids from the identified peptides having confidence greater than or equal to 95% divided by the total number of amino acids in the sequence. Several quantitative estimates provided for each protein by ProteinPilot were utilized: the fold change ratios of differential expression between labelled protein extracts; the p-value, representing the probability that the observed ratio is different than 1 by chance. A decoy database search strategy was also used to estimate the false discovery rate (FDR), defined as the percentage of decoy proteins identified against the total protein identification. The FDR was calculated by searching the spectra against the decoy database generated from the target database. The results were then exported into Excel for manual data interpretation. Although relative quantification and statistical analysis were provided by the ProteinPilot software, an additional 1.3-fold change cutoff for all iTRAQ ratios (ratio <0.77 or >1.3) and a p-value lower tan 0.05 were selected to classify proteins as up- or down-regulated (at least in two of three biological replicates). Proteins with iTRAQ ratios below the low range (0.77) were considered to be underexpressed, whereas those above the high range (1.3) were considered to be overexpressed.

### Data analysis for nuclear proteomes

The peptide quantification was performed using the Progenesis LC−MS software (ver. 2.0.5556.29015, Nonlinear Dynamics). Using the accurate mass measurements from full survey scans in the TOF detector and the observed retention times, runs were aligned to compensate for between-run variations in our nanoLC separation system. To this end, all runs were aligned to a reference run automatically chosen by the software, and a master list of features considering m/z values and retention times was generated. The quality of these alignments was manually supervised with the help of quality scores provided by the software. The peptide identifications were exported from Protein Pilot software and imported in Progenesis LC− MS software where they were matched to the respective features. Output data files were managed for subsequent statistical analyses and representation. Proteins identified by site (identification based only on a modification), reverse proteins (identified by decoy database) and potential contaminants were filtered out. Proteins quantified with at least two unique peptides, a *T*-test p-value lower than 0.05, and an absolute fold change of <0.77 (down-regulation) or >1.3 (up-regulation) in linear scale were considered significantly differentially expressed.

### Bioinformatics

The proteomic data were analyzed using QIAGEN’s Ingenuity^®^ Pathway Analysis (IPA) (QIAGEN Redwood City, www.qiagen.com/ingenuity), to detect and infer differentially activated/deactivated pathways because of Delta-24RGD treatment. This software comprises curated information from databases of experimental and predictive origin, enabling discovery of highly represented functions, pathways, and interactome networks. The IPA comparison analysis considers the signalling pathway rank according to the calculated p-value and reports it hierarchically. The software generates significance values (p-values) between each biological or molecular event and the imported molecules based on the Fisher’s exact test (p ≤ 0.05).

### Secretome analysis

A dot-blot protein array was used for cytokine profiling (Abcam). Briefly, membranes with 80 cytokine antibodies were blocked with the manufacturer’s blocking buffer at room temperature (RT) for 30 min, and incubated o/n with 1ml of undiluted cell-cultured media from Mock- and U87-infected cells (3, 6, and 10hpi) (n=3). After washing, a biotinylated anti-cytokine antibody mixture was added to the membranes followed by incubation with HRP-conjugated streptavidin and then exposed to the manufacturer’s peroxidase substrate. Chemiluminescence signals were quantified with the ImageQuant ECL system (BioRad) and normalized to the background levels and positive control signals. The Perseus software (version 1.5.6.0) was used for statistical analysis [63].

### Western-blotting

Equal amounts of protein (10 μg) were resolved in 4–15% Criterion™ TGX Stain-Free™ Protein Gels (#5678085 Bio-rad). Mock-infected and U87-infected protein cell extracts were electrophoretically transferred onto nitrocellulose membranes using Trans-Blot Turbo (BioRad) for 7 minutes at 2.5A constant, up to 25V. Equal loading of the gels was assessed by stain free digitalization and Ponceau staining. Membranes were probed with primary antibodies at 1:1000 dilution in 5% nonfat milk or BSA. After incubation with the appropriate horseradish peroxidase-conjugated secondary antibody (1:5000), antibody binding was detected by a Chemidoc™MP Imaging System (Bio-Rad) after incubation with an enhanced chemiluminescence substrate (Perkin Elmer). All Band intensities were measured with Image Lab Software Version 5.2 (Bio-Rad) and normalized to GAPDH.

## CONCLUSIONS

From a biological point of view, we have partially deciphered the molecular events triggered by Delta-24-RGD prior to the induction of autophagy in glioma cells. Moreover, we have established a working pipeline for the future monitorization of the intracellular and extracellular proteostatic derangements induced by Delta-24-RGD-based vectors in pre-clinical glioma models. The high-throughput, and straightforward workflow applied in this study has allowed us the generation of quantitative molecular maps that may be useful to the development of complementary adenoviral based-vectors to increase the specificity and potency against glioma. Finally, we consider that the evaluation of the cytokine panel modulated by Delta-24-RGD might be of potential clinical interest, with the aim to monitoring the immune response elicited by Delta-24-RGD treatment in patients that participate in clinical trials where this therapeutic vector is being supplied.

## SUPPLEMENTARY MATERIALS FIGURES AND TABLES







## Abbreviations

GBM: glioblastoma
STAT3: Signal transducer and activator of transcription 3
PKC: Protein kinase C
ERK1/2: extracellular signal–regulated kinase 1/2
p38 MAPK: p38 mitogen-activated protein kinase
c-JUN: Transcription factor AP-1
