# Supplementary material for: Spatial and temporal proteome dynamics of glioma cells during oncolytic adenovirus Delta-24-RGD infection
Source: Oncotarget. 2018 Jul 24;9(57):31045–65. doi: 10.18632/oncotarget.25774 (PMC6089549; doi:10.18632/oncotarget.25774)
Supplement: Supplementary file 1 [file oncotarget-09-31045-s001.pdf]

Spatial and temporal proteome dynamics of glioma cells during oncolytic adenovirus Delta-24-RGD infection

SUPPLEMENTARY MATERIALS

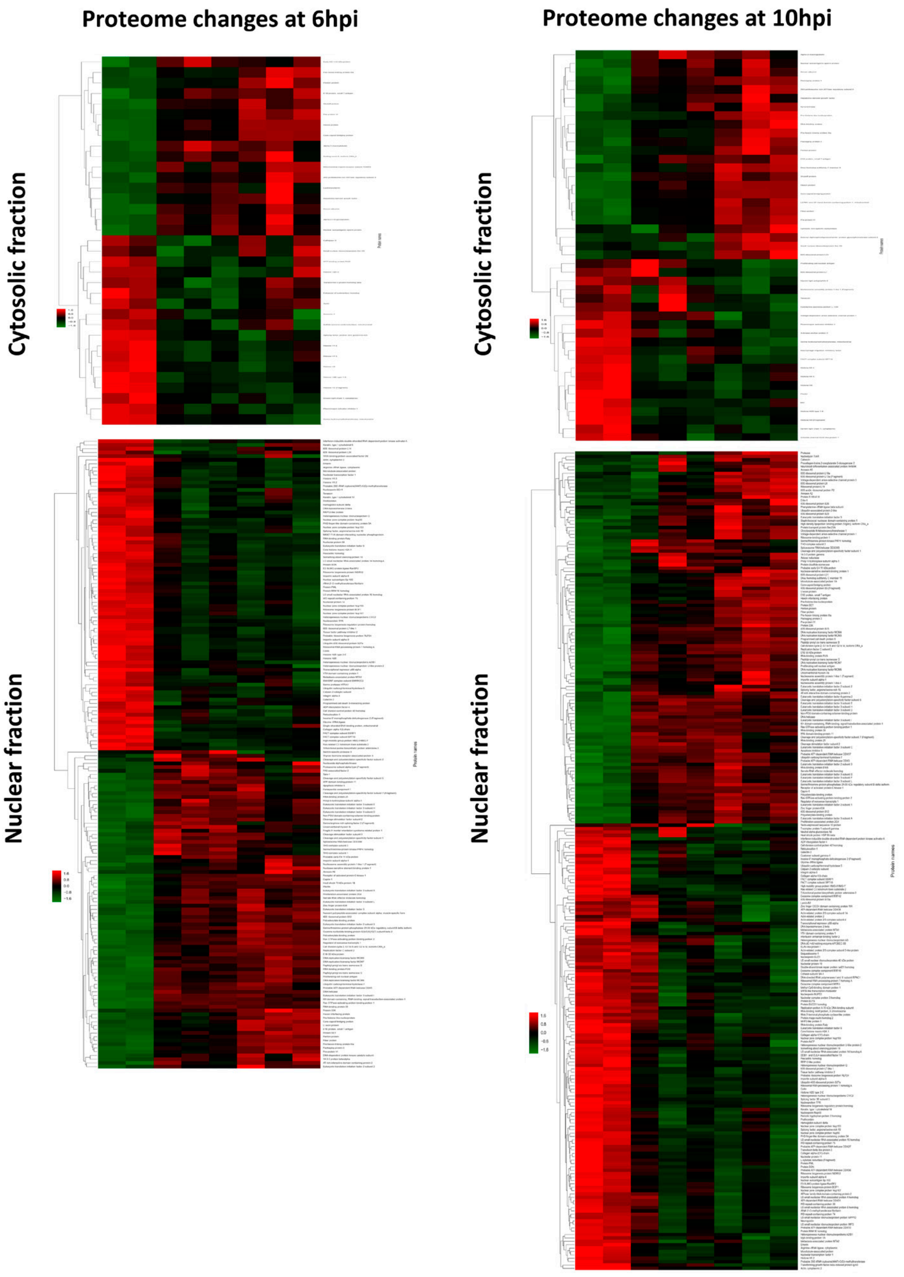

Supplementary Figure 1: Heat maps representing the degree of change for the differentially expressed proteins (P < 0.05) upon Delta-24-RGD infection (early time points). Lateral Legend indicates color-coded fold-change on Log10 scale. Green (down), and up- (red) regulated proteins respect to Mock cells.

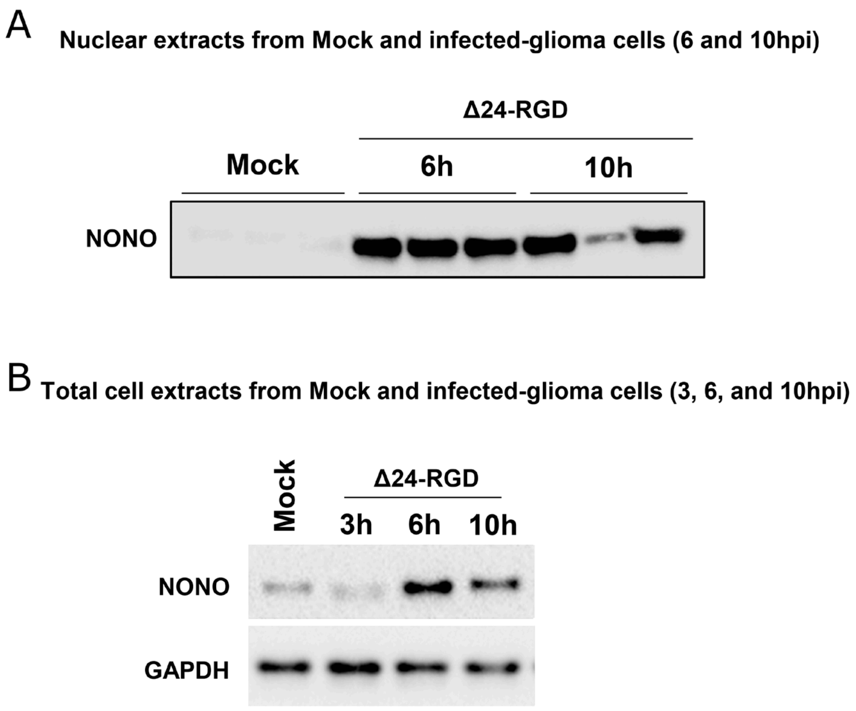

Supplementary Figure 2: Modulation of NONO expression during Delta-24-RGD infection. Western blotting analysis were performed in nuclear extracts **(A)** and also in independent total cells extracts **(B)** from infected and Mock glioma cells. Our results confirm the increment of the transcriptional regulator NONO protein levels during the infection, partially validating the LC-MS/MS approach used in this study.

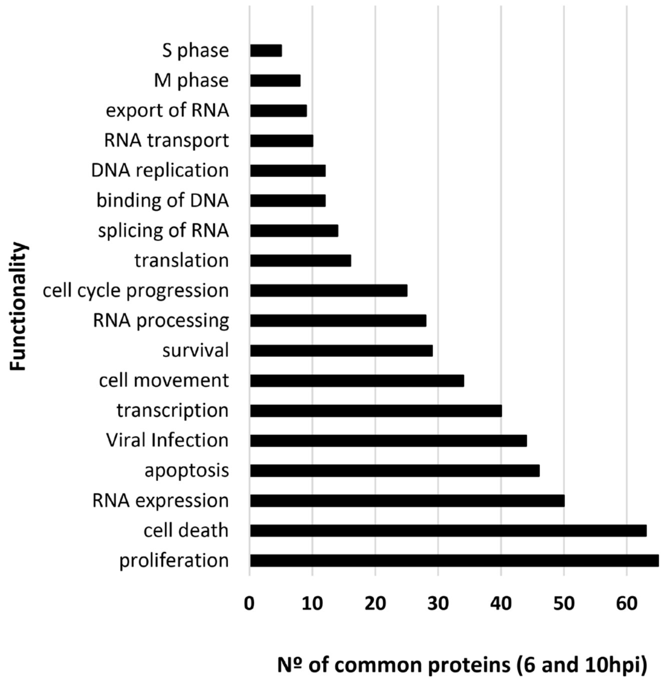

Supplementary Figure 3: Molecular functions significantly represented in the common proteome differentially expressed upon Delta-24-RGD infection at 6 and 10hpi. Highly significant terms ($p<0.001$) are shown.

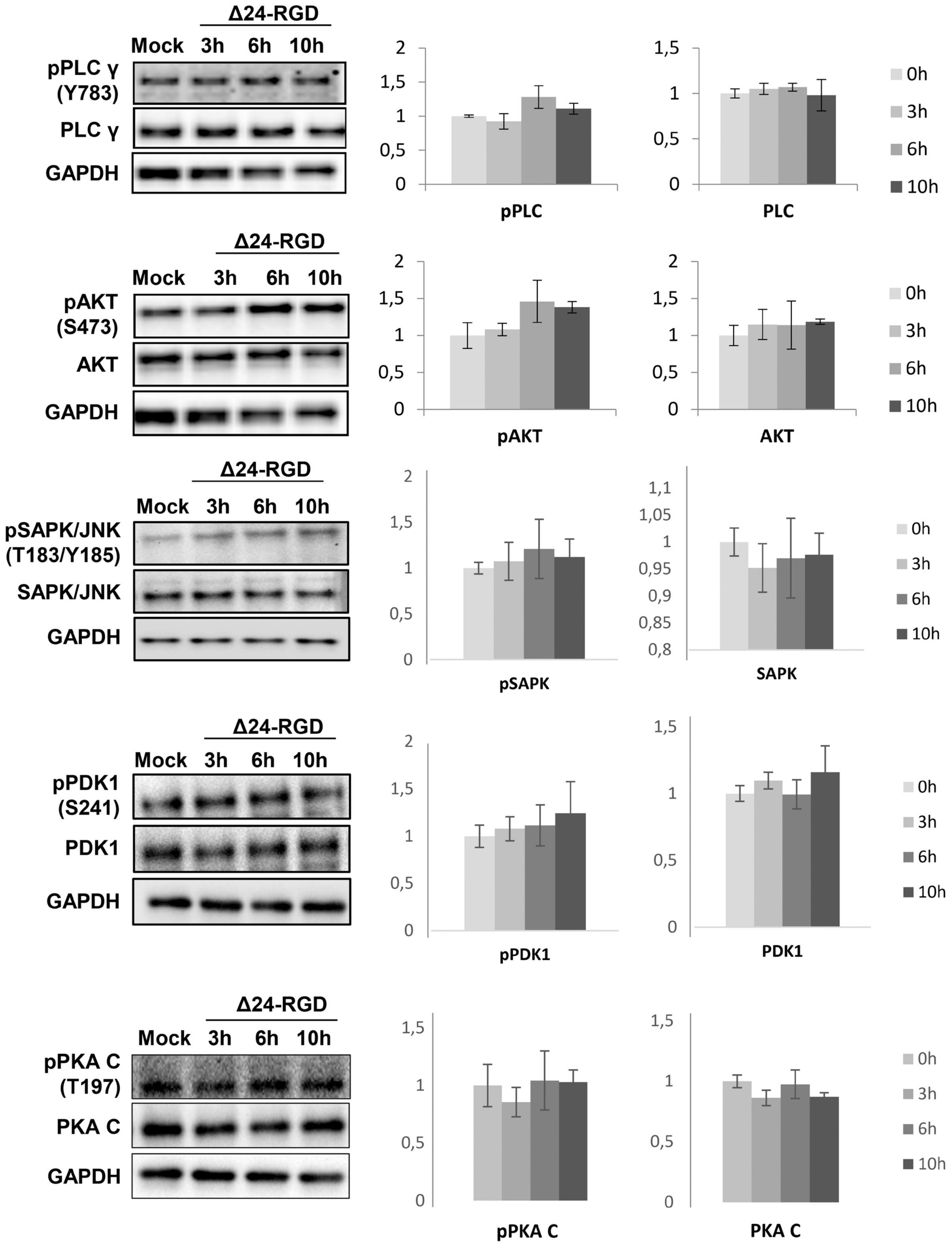

Supplementary Figure 4: Activation status of specific survival kinases during Delta-24-RGD infection. No significant changes were detected for this kinase panel. Experiments were performed in independent biological triplicates.

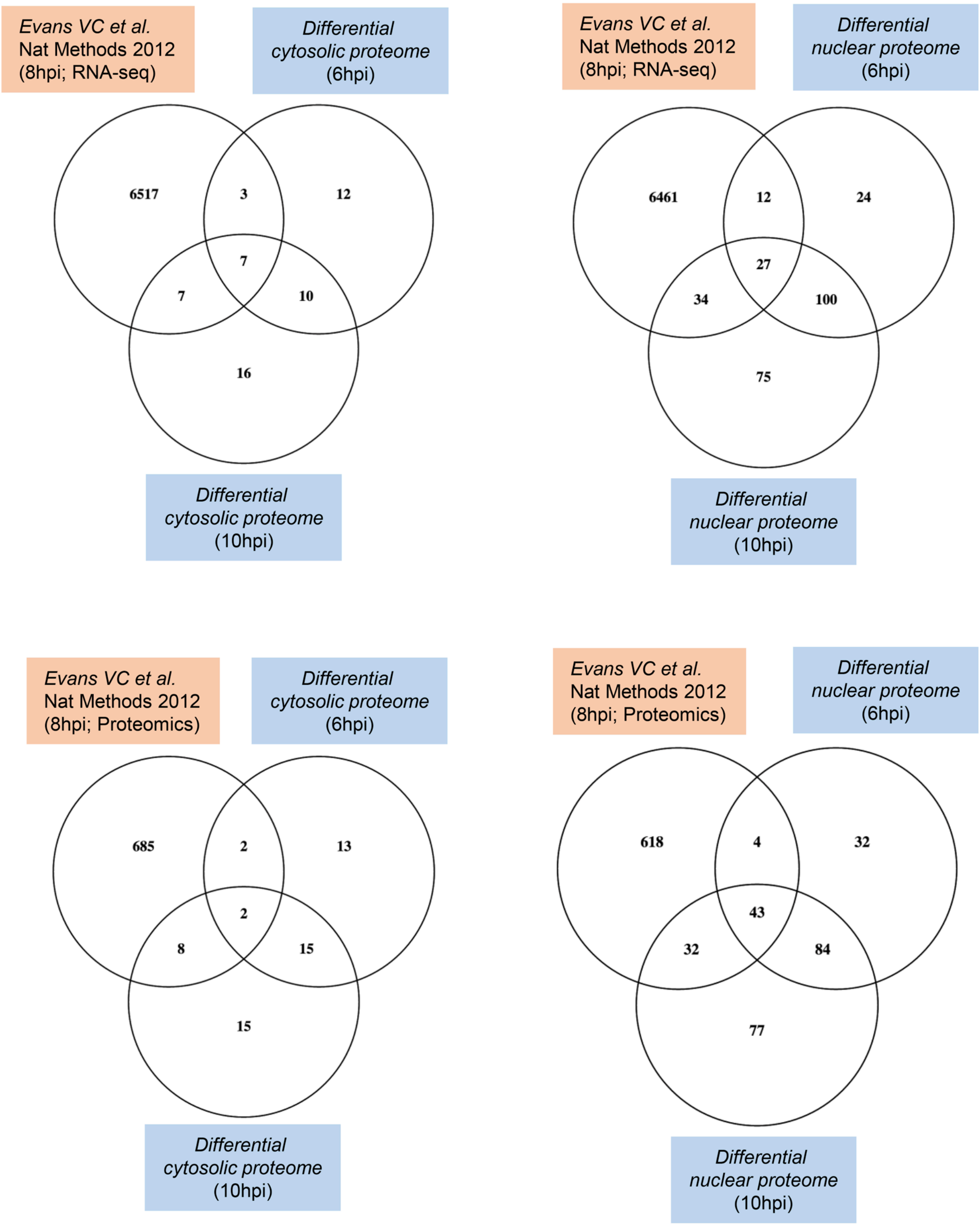

Supplementary Figure 5: Overlap between this study and the study performed by Evans VC et al. Molecular fingerprints obtained in both studies were compared.

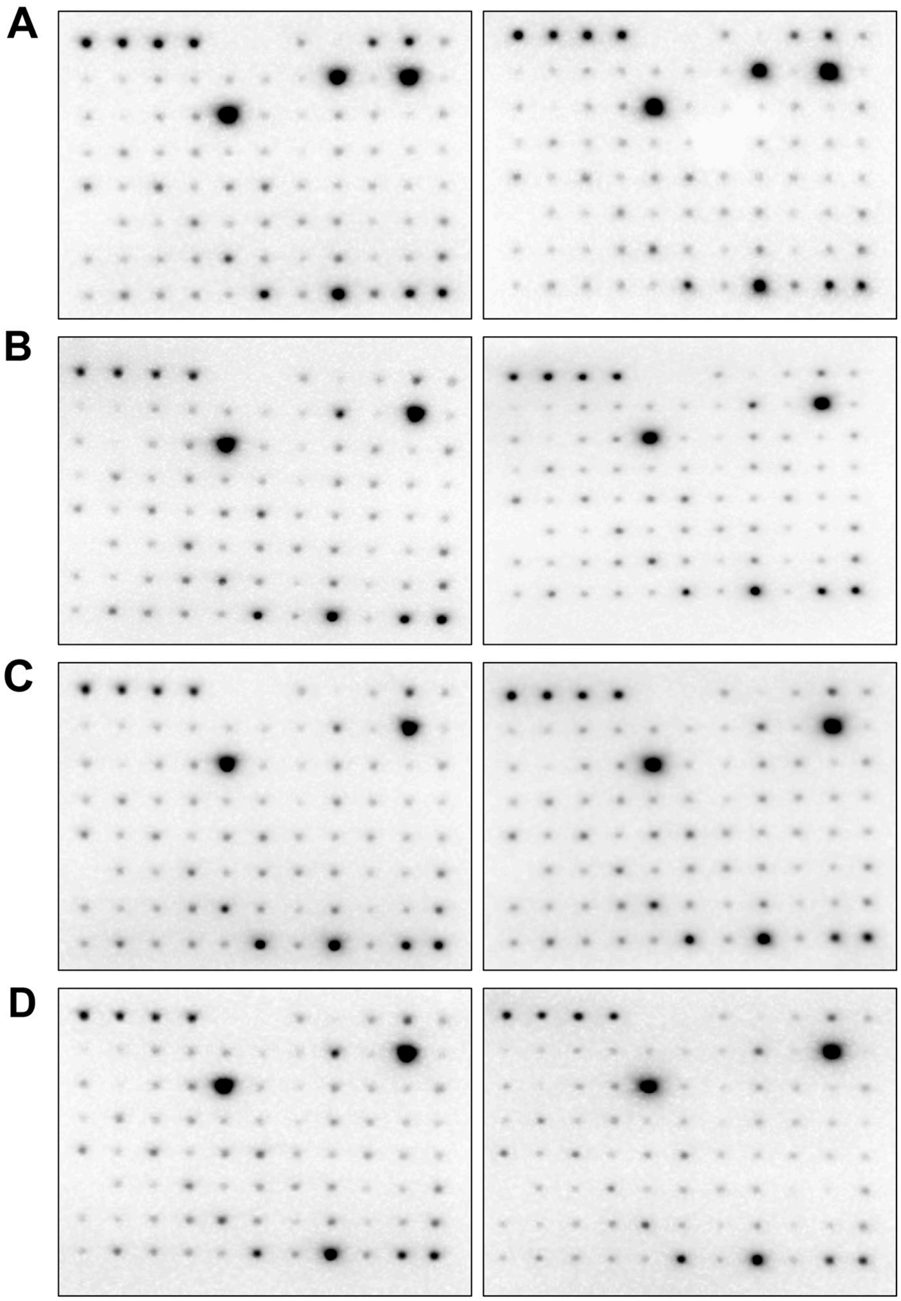

Supplementary Figure 6: Representative images of the cytokine arrays.

Supplementary Table 1: Nuclear proteome alterations in Delta24RGD-infected cells.

See Supplementary File 1

Supplementary Table 2: Cytosolic proteome alterations in Delta24RGD-infected cells.

See Supplementary File 2
